# Influence of parental behavior on myopigenic behaviors and risk of myopia: analysis of nationwide survey data in children aged 3 to 18 years

**DOI:** 10.1186/s12889-022-14036-5

**Published:** 2022-08-30

**Authors:** Yao-Lin Liu, Jia-Pang Jhang, Chuhsing Kate Hsiao, Tzu-Hsun Tsai, I-Jong Wang

**Affiliations:** 1 Department of Ophthalmology, National Taiwan University Hospital, College of Medicine, National Taiwan University, Taipei, Taiwan; 2 Institute of Epidemiology and Preventive Medicine, College of Public Health, National Taiwan University, Taipei, Taiwan

**Keywords:** Parental behavior, Family, Elementary school, Kindergarten, Myopia, High myopia, High school, Near work activities

## Abstract

**Background:**

Preventive parental behavior may play an important role in the outcomes of children’s myopia. We investigated associations between parental behavior and children’s myopia status and daily activities using data from the most recent myopia survey in Taiwan.

**Methods:**

In total, 3845 children aged 3 to 18 years who completely responded to the questionnaire were included (total score ranging from 0 to 75). A score of ≥ 50 was considered to indicate beneficial parental behavior. Time allocation data for near-work activities, using electronic devices, and outdoor activities were collected using a separate self-reported questionnaire. Associations between beneficial parental behavior and children’s myopia status and activity patterns were analyzed and stratified by school level.

**Results:**

Beneficial parental behavior was positively associated with children’s myopia in the overall samples [adj. odds ratio (OR): 1.31, 95% confidence interval (CI): 1.08–1.59, *p* = 0.006)] and at the elementary school level (adj. OR: 1.43, 95% CI: 1.11–1.83, *p* = 0.005). However, a negative association with high myopia was observed in the overall samples (adj. OR: 0.71, 95% CI: 0.50–0.99, *p* = 0.049) and high school level (adj. OR: 0.62, 95% CI: 0.41–0.92, *p* = 0.02). Beneficial parental behavior was associated with less time spent on near work (≥ 180 min/day) and electronic device use (≥ 60 min/day), but not with outdoor activities.

**Conclusion:**

In Taiwan, children’s myopia is associated with higher rate of parents’ beneficial behaviors, which suggests that regular vision surveillance is necessary to promote better parental behavior toward children’s eye care. Certain parental practices may influence children’s behavior pattern and reduce the risk of children’s high myopia development in the long run.

**Supplementary Information:**

The online version contains supplementary material available at 10.1186/s12889-022-14036-5.

## Background

The worldwide prevalence of myopia has been steadily increasing for several decades [1, 2], and its rising prevalence and magnitude in East Asian countries have reached epidemic proportions [3, 4]. With pathologic changes that develop along with excessive axial elongation of the eyeball at an early age, people with high myopia have a lifelong increased risk of a wide variety of ocular diseases, including early cataracts, retinal detachment, glaucoma, and myopic macular degeneration [5]. Therefore, high myopia is also considered pathologic or degenerative myopia, which is one of the leading causes of blindness among young and middle-aged adults in Asia [4].

In Taiwan, the National Taiwan University Hospital conducted eight nationwide serial surveys of myopia in schoolchildren from 1983 to 2017. The data demonstrated a clear trend of an increasing rate of myopia among children at varying school levels [6]. The prevalence of high myopia among adolescents almost tripled over the recent 30-year period. The eighth survey, conducted between 2016 and 2017, also clarifies the impact of educational pressure-associated near-work activities and the use of electronic devices on myopia development [6]. Therefore, interventions targeting behavior modification are of paramount importance.

A school-based approach is a straightforward strategy for executing public health policies for myopia prevention and control. These approaches usually involve implementing enhanced health education and increased time outdoors [2, 7]. In addition to school-based intervention, another potential approach to modify children’s behavior through their families has been previously explored least out of all approaches. Parental influence on modifying children’s behavior has been recognized to prevent childhood overweight or obesity by encouraging a healthy diet intake, increasing physical activities, and reducing sedentary screen time [8–11]. However, studies on the association between parental behavior and children’s refractive status and the potential parental role in myopia prevention and control are scarce [12, 13]. Although these studies showed the beneficial effect of parental behavior on children’s vision care, they are limited by the narrow age range of the study subjects, the lack of accurate refractive measurement, and the lack of focused analysis on high myopia, which represents the long-term outcome of parental influence.

This study aimed to explore associations between parental behaviors and children’s myopia through analyzing questionnaires from a whole population-based survey.

## Materials and methods

### Participants

This study was conducted using data derived from the most recent myopia survey of children from 2016 to 2017. The detailed research method and overall results of the estimated prevalence of myopia in Taiwan have been thoroughly described in a previously published study [6]. The target population included children between 3 and 18 years of age, including kindergarten, elementary school, junior high school, and senior high/vocational school. Probability proportional to size sampling with stratification by three urbanization levels was utilized to sample the target population. Cycloplegic refraction examinations were performed in all participating children. Demographic information, parental behavior toward myopia prevention and control, and personal activity patterns were collected using a questionnaire answered by both children and their parents. Overall, 7348 children completed cycloplegic refraction in the 2016 survey, with a response rate of 76.48%. To analyze the association between parental behavior and children’s myopia, we extracted information about parental behavior and associated covariates, including parental education level, parental refractive status, parental smoking habits, and socioeconomic status (SES) based on family income from the questionnaire data. In total, 3845 parents (52.3%) completed these parts of the questionnaire. There was no difference in age, rate of myopia and high myopia between the response (*n* = 3845) and non-response group (*n* = 3503). Except for a slightly male dominance in non-response group at high school level (59.5% vs. 52.9%, *p* = 0.001).

### Parental behavior toward myopia prevention and control

The original questionnaire is available in the online supplement of our previously published paper (https://www.aaojournal.org/article/S0161-6420(20)30679-5/fulltext” \l “supplementaryMaterial”). In total, 15 questions were designed. Each corresponding answer was scored from 0 to 5 points based on six levels, with higher scores indicating myopia control. Hence, the total score ranged from 0 to 75 points. The third quartile was 50 points, with a score above 50 indicating beneficial parental behavior. In addition to the behavior score, parental attitudes toward children’s extracurricular timetables, including time allocation for outdoor activities, reading, electronic device use, and cram school classes, were also documented. Questions regarding parental attitudes toward cram school were removed from the questionnaire if the subjects were at the kindergarten level. No points were assigned for orthokeratology questions when counting the total parental behavior score in kindergarten children.

### Covariates of parental data

Self-reported myopia in at least one of the parents was defined as parental myopia. Parental education level was considered high if either parent had completed graduate studies. SES was categorized as high if self-reported monthly family disposable income was above 75,000 New Taiwan Dollars (2700 United States dollars), which was above the medium of household disposable income of 73,865 New Taiwan Dollars (2660 United States dollars) in 2017. Self-reported smoking in at least one of the parents was defined as positive for the covariate of parental smoking. Parental myopia, high parental education level, and high SES were all associated with beneficial parental behavior (behavior score ≥ 50) (parental myopia, crude odds ratio [OR]: 1.90; high parental education level, crude OR: 1.70; high SES, crude OR: 1.29; all *P* values < 0.001). These covariates were treated as confounders and adjusted in the subsequent multivariate analyses.

### Children’s refractive status

Cycloplegic refraction of the right eye was used as the major response variable, measured with an autorefractor and retinoscopy 30 min after administering three drops of 0.5% tropicamide at 5-min intervals. Myopia was defined as a spherical equivalent (SE) ≤ -0.5 D (Diopter), and high myopia was defined as SE ≤ -5.0 D. Myopic children were classified into mild (-2.0 D < SE ≤ -0.5 D), moderate (-5.0 D < SE ≤ -2.0 D), and high (SE ≤ -5.0 D) myopia, which facilitated further exploration of the relationship between parental behavior and children’s refractive status.

### Time spent on daily activities

Information about children’s activity patterns was also extracted from the self-reported questionnaire data outlined in a previous study [6]. The activity comprised the duration of outdoor and near-work activities. Information about the duration of computer, smartphone, and tablet use was also obtained and included in calculating total near-work time. Near work time ≥ 180 min/day was categorized as excessive, electronic devices use time ≥ 60 min/day was categorized as excessive, and outdoor activity time ≥ 60 min/day was categorized as adequate. The associations between parental behavior and children’s time spent on near-work activities, electronic device use, and outdoor activities were analyzed to understand the possible influences of parental behavior on children’s activities.

### Data analysis

The previous analysis of the refractive status of children shows that myopia distribution varies widely among different school levels. Therefore, all analyses in this study were performed not only in the overall samples and in each stratification of school level (i.e., kindergarten, elementary school, and high school). The chi-square test and Student’s t-test were used to compare categorical variables and continuous variables, respectively. Univariate and multivariate logistic regressions were used to calculate the crude and adjusted OR of myopia, high myopia, and each category of children’s activity. In addition to the analyses of the total parental behavior score, further analyses using principal component analyses (PCA) were conducted to understand the detailed patterns of parental behavior. All individual questions in the questionnaire on parental behavior and parental attitudes toward children’s extracurricular time allocation were included. The number of principal components was selected based on the turning point of the scree plot. The weighting of all questions on each component was visualized on a heatmap. R statistical software, version 4.0.0 (R Foundation Inc., Vienna, Austria), was used for all statistical analyses, and all reported *P* values were 2-sided with a significance level of 0.05.

### Ethics statement

All surveys followed the tenets of the Declaration of Helsinki. The study protocol, recruitment method, and consent procedure were approved by the research ethics committees of the National Taiwan University Hospital (ClinicalTrials.gov identifier: NCT03750630). Written informed consent was obtained prospectively from the participants and their parents or guardians.

## Results

### Demographic data of the study population

A descriptive analysis of the children’s demographic information is shown in Table 1. Of 3845 subjects, 1894 (49.2%) had myopia. Myopia rates differed among different school levels as follows: 5.3% in kindergarten, 42.9% in elementary school, and 82.4% in high school. Overall, parents of myopic children had a lower rate of high education (14.1% vs. 17.4%, *P* = 0.008), lower behavior scores (44.30 ± 7.50 vs 46.31 ± 7.90%, *P* < 0.001), and lower rates of beneficial behavior (23.8% vs. 34.6%, *P* < 0.001) than parents of non-myopic children. There was no significant difference in demographic profiles and parental behavior scores between myopic and non-myopic children in kindergarten.

**Table 1:** Demographic data of the study population, overall and stratified by school level

	**Overall**			**Kindergarten**			**Elementary school**			**High school**		
	**(*N* = 3845)**			**(*n* = 950)**			**(*n* = 1374)**			**(*n* = 1521)**		
	**Myopia**	**No myopia**	***p* value**	**Myopia**	**No myopia**	***p* value**	**Myopia**	**No myopia**	***p* value**	**Myopia**	**No myopia**	***p* value**
**Number of children**	1894	1951		50	900		590	784		1254	267	
**Age (mean ± SD)**	13.47 ± 3.34	7.92 ± 3.66	** < 0.001**	4.94 ± 0.89	4.95 ± 0.85	0.95	9.99 ± 1.72	8.88 ± 1.75	** < 0.001**	15.45 ± 1.71	15.04 ± 1.79	** < 0.001**
**Male (%)**	971 (51.3)	1012 (51.9)	0.72	24 (48.0)	471 (52.3)	0.65	291 (49.3)	392 (50.0)	0.83	656 (52.3)	149 (55.8)	0.33
**High SES (%)**	725 (38.3)	722 (37.0)	0.26	14 (28.0)	336 (37.3)	0.19	258 (43.7)	295 (37.6)	**0.03**	453 (36.1)	91 (34.1)	0.37
**Parental smoking (%)**	777 (41.0)	790 (40.5)	0.70	20 (40.0)	349 (38.8)	0.93	240 (40.7)	317 (40.4)	0.97	517 (41.2)	124 (46.4)	0.11
**High parental education level (%)**	267 (14.1)	339 (17.4)	**0.008**	7 (14.0)	155 (17.2)	0.73	111 (18.8)	153 (19.5)	0.81	149 (11.9)	31 (11.6)	0.94
**Parental myopia (%)**	1330 (70.2)	1381 (70.8)	0.61	41 (82.0)	672 (74.7)	0.11	486 (82.4)	581 (74.1)	**0.001**	803 (64.0)	128 (47.9)	** < 0.001**
**Parental behavior score (mean ± SD)**	44.30 ± 7.50	46.31 ± 7.90	** < 0.001**	48.24 ± 6.42	48.30 ± 7.52	0.95	47.17 ± 7.69	45.78 ± 7.50	** < 0.001**	42.78 ± 6.98	41.12 ± 7.75	**0.001**
**Beneficial parental behaviour (score≧50) (%)**	450 (23.8)	676 (34.6)	** < 0.001**	22 (44.0)	396 (44.0)	1.00	223 (37.8)	243 (31.0)	**0.01**	205 (16.3)	37 (13.9)	0.36

Socioeconomic status was defined as high if self-reported monthly family income was above 75,000 New Taiwan Dollars

Parental smoking was defined as self-reported smoking habit in either parent or both

Parental education level was defined as high if either parent had completed a graduate school

Parental myopia was defined as self-reported myopia in either parent or both

*SD* Standard deviation, *SES* Socioeconomic status

Number in bold indicated *p* < 0.05

In elementary school, myopic children had a higher rate of high family SES (43.7% vs. 37.6%, *P* = 0.03) than non-myopic children. Parents of myopic children had higher rates of myopia (82.4% vs. 74.1%, *P* = 0.001), higher behavior scores (47.17 ± 7.69 vs. 45.78 ± 7.50, *P* < 0.001), and higher rates of beneficial behavior (37.8% vs. 31.0%, *P* = 0.01) than parents of non-myopic children. In high school, parents of myopic children had higher rates of myopia (64.0% vs. 47.9%, *P* < 0.001) and higher behavior scores (42.78 ± 6.98 vs. 41.12 ± 7.75, *P* = 0.001) than parents of non-myopic children; however, there was no significant difference in the rate of beneficial behavior (16.3% vs. 13.9%, *P* = 0.36).

### The distribution of beneficial parental behavior by children’s refractive status

**Fig. 1:**
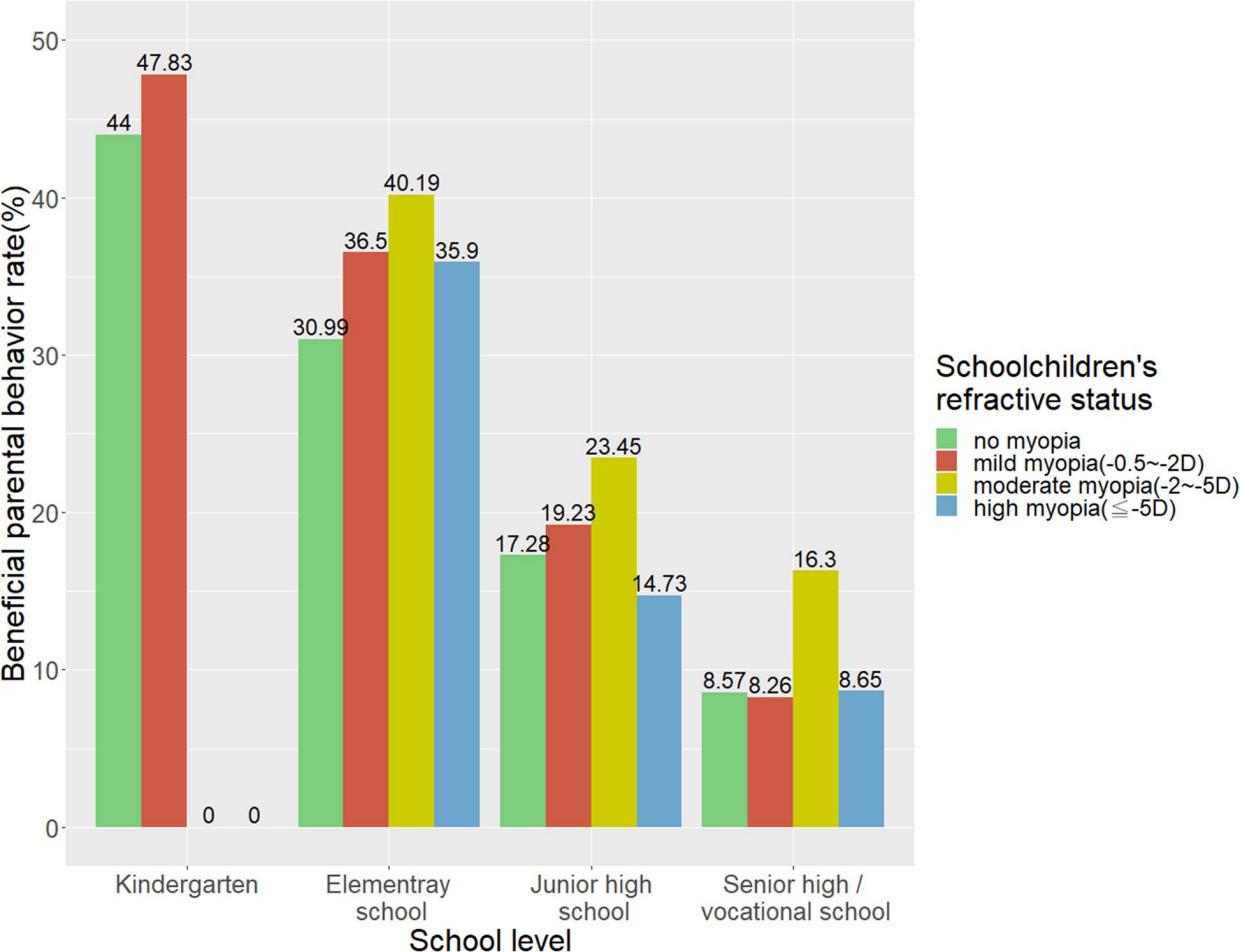
Beneficial parental behavior rate (behavior score ≥ 50) in different school levels, stratified by schoolchildren's refractive status

Figure 1 illustrates the rate of beneficial parental behavior (behavior score ≥ 50) at different school levels stratified by children’s refractive status. There was a clear trend that the higher the school level, the lower the rate of beneficial parental behavior in myopia control. The highest rate was consistently observed at each school level in the subgroup of children with moderate myopia. While gross comparison of the overall samples showed that myopic children had a significantly lower rate of beneficial parental behavior, this association was largely confounded by age or school levels in essence, because a higher beneficial parental behavior rate tended to be observed at the young age group, wherein the myopia rate was low. Therefore, analyses under the stratification of school level were crucial to clarify the unconfounded association between parental behavior and children’s myopia.

### The associations between beneficial parental behavior and children’s myopia and high myopia

The results of univariate and multivariate logistic regression analyses of the association between beneficial parental behavior (behavior score ≥ 50) and children’s myopia and high myopia are shown in Table 2. Age, parental myopia, parental education level, and SES were adjusted in the multivariate models. A significant positive association between beneficial parental behavior and children’s myopia was identified in the overall samples (adjusted [adj.] odds ratio [OR]: 1.31, 95% confidence interval [CI]: 1.08–1.59, *P* = 0.006) and at the elementary school level (adj. OR: 1.43, 95% CI: 1.11–1.83, *P* = 0.005). However, a significant negative association between beneficial parental behavior and children’s high myopia was observed in the overall samples (adj. OR: 0.71, 95% CI: 0.50–0.99, *P* = 0.049) and high school level (adj. OR: 0.62, 95% CI: 0.41–0.92, *P* = 0.02). To summarise our findings, a higher rate of beneficial parental behavior was associated with children’s myopia, especially at the elementary school level; additionally, a lower rate of beneficial parental behavior was associated with children’s high myopia, especially at the high school level.

**Table 2:** Associations between rate of beneficial parental behavior (behavior score≧50) and schoolchildren’s myopia (2A) and high myopia (2B), overall and stratified by school level

**2A**							
		**Myopia (≤ -0.5D)**	**No myopia**	**Crude OR (95% CI)**	***p* value**	**Adj. OR (95% CI)**	***p* value**
**Overall *n* = 3845**	Number	1894	1951	0.59 (0.51, 0.69)	** < 0.001**	1.31 (1.08, 1.59)	**0.006**
	Rate	23.8%	34.6%				
**Kindergarten *n* = 950**	Number	50	900	1.0 (0.54, 1.84)	1.0	1.06 (0.57, 1.93)	0.85
	Rate	44.0%	44.0%				
**Elementary school *n* = 1374**	Number	590	784	1.35 (1.07, 1.70)	**0.01**	1.43 (1.11, 1.83)	**0.005**
	Rate	37.8%	31.0%				
**High school *n* = 1521**	Number	1254	267	1.21 (0.83,1.83)	0.36	1.20 (0.81, 1.81)	0.29
	Rate	16.3%	13.9%				
**2B**							
		**High Myopia (≤ -5D)**	**No high myopia**	**Crude OR (95% CI)**	***p* value**	**Adj. OR (95% CI)**	***p* value**
**Overall *n* = 3845**	Number	378	3467	0.35 (0.25, 0.47)	** < 0.001**	0.71 (0.50, 0.99)	**0.049**
	Rate	13.5%	31.0%				
**Kindergarten *n* = 950**	Number	2	948	NA	NA	NA	NA
	Rate	0%	44.1%				
**Elementary school *n* = 1374**	Number	39	1335	1.09 (0.52, 2.21)	0.86	1.29 (0.64, 2.52)	0.47
	Rate	35.9%	33.9%				
**High school *n* = 1521**	Number	337	1184	0.59 (0.39, 0.86)	**0.005**	0.62 (0.41, 0.92)	**0.02**
	Rate	11.0%	17.3%				

Number in bold indicated *p* < 0.05

Age, parental myopia, parental education level and family socioeconomic status were adjusted in multivariate logistic regression models

*OR* Odds ratio, *CI* Confidence interval, *NA* Not available

### The associations between beneficial parental behavior and children’s activity time

The associations between parental behavior and children’s time spent on near-work activities, electronic device use, and outdoor activities are shown in Table 3. Overall, beneficial parental behavior was associated with less time spent on near work activities (≥ 180 min/day, adj. OR: 0.79, *P* = 0.01) and less time on electronic device use (≥ 60 min/day; adj. OR, 0.48, *P* < 0.001) but not with time on outdoor activities. In stratified analysis by school level, the association with near work time was observed in kindergarten, but not in elementary and high school; additionally, the association with time on electronic device use was observed in kindergarten and elementary school, but not in high school.

**Table 3:** Associations between beneficial parental behavior (behavior score ≥ 50) and schoolchildren’s reported time of different activities, overall and stratified by school level

		**Overall**		**Kindergarten**		**Elementary school**		**High school**	
**Children’s activity**		OR (95% CI)	*p* value	OR (95% CI)	*p* value	OR (95% CI)	*p* value	OR (95% CI)	*p* value
**Excessive nearwork time (≥ 180 min/day)**	Crude	0.54 (0.46, 0.63)	** < 0.001**	0.29 (0.18, 0.45)	** < 0.001**	0.93 (0.69, 1.26)	0.65	1.57 (1.06, 2.37)	**0.02**
	Adjusted	0.79 (0.66, 0.95)	**0.01**	0.30 (0.19, 0.46)	** < 0.001**	0.93 (0.69,1.27)	0.67	1.35 (0.91, 2.06)	0.14
**Excessive electronic devices using time (≥ 60 min/day)**	Crude	0.35 (0.29, 0.42)	** < 0.0001**	0.23 (0.15, 0.34)	** < 0.001**	0.45 (0.33, 0.60)	** < 0.001**	0.90 (0.64, 1.27)	0.56
	Adjusted	0.48 (0.40, 0.58)	** < 0.0001**	0.24 (0.16, 0.35)	** < 0.001**	0.45 (0.33, 0.62)	** < 0.001**	0.90 (0.64, 1.27)	0.54
**Adequate outdoor activity time (≥ 60 min/day)**	Crude	0.97 (0.82, 1.15)	0.74	0.81 (0.61, 1.08)	0.15	1.19 (0.89, 1.60)	0.22	1.10 (0.77, 1.58)	0.66
	Adjusted	0.97 (0.81,1.16)	0.74	0.76 (0.57, 1.02)	0.07	1.20 (0.90, 1.61)	0.22	0.97 (0.68, 1.40)	0.87

Electronic devices using time was included in the calculation of the near work time

Age, parental myopia, parental education level and family socioeconomic status were adjusted in multivariate logistic regression models

*OR* Odds ratio, *CI* Confidence interval

Number in bold indicated *p* < 0.05

### Principal component analysis of the questionnaire about parental behavior

**Fig. 2:**
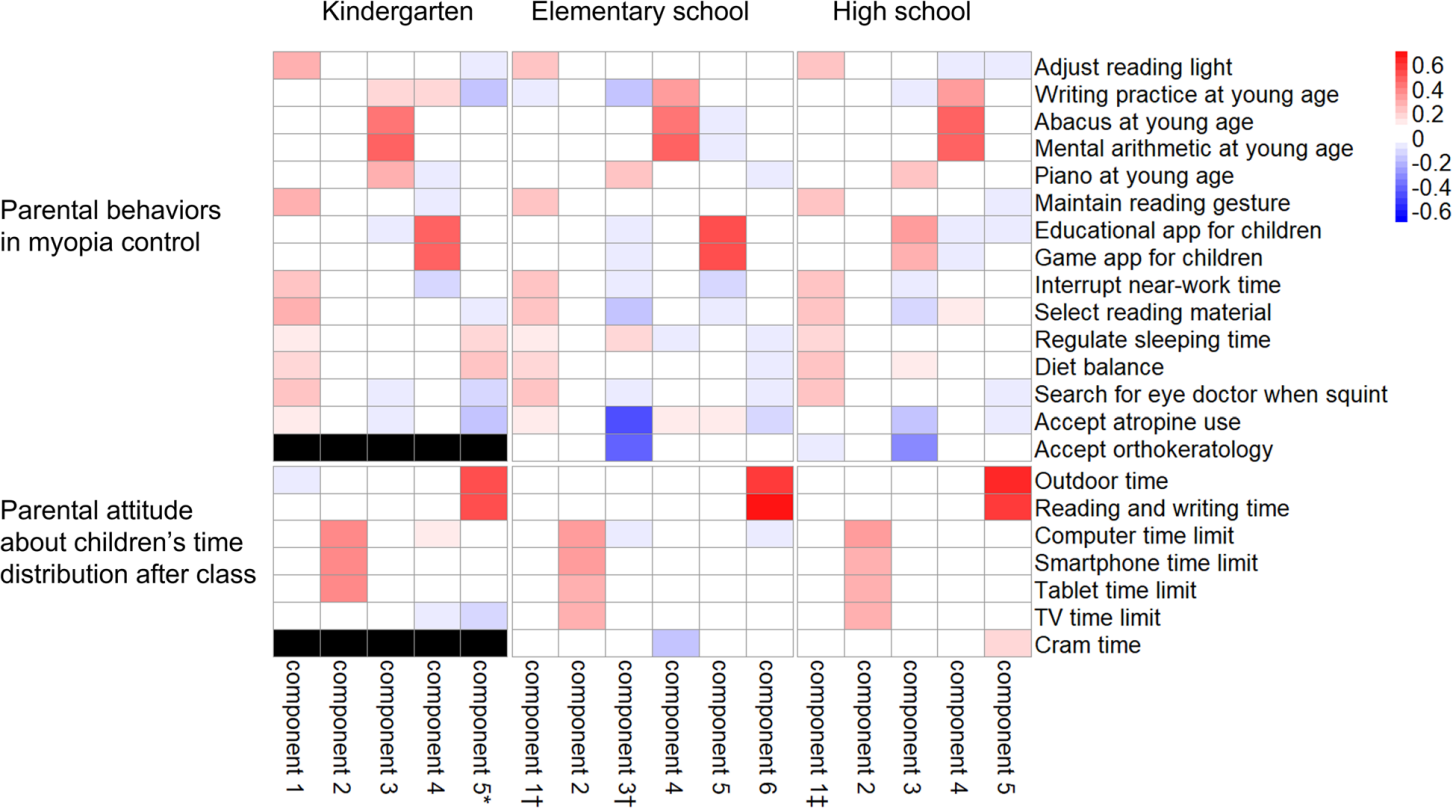
Heatmap of the weightings of all questions contributing to each component from principal component analyses in kindergarten, elementary school, and high school, respectively. All questions in the questionnaire on parental behavior in myopia control and parental attitude about children’s time distribution after class were included. Cells in black indicate that these questions were not asked at the kindergarten level. Multivariate logistic regression analyses for children’s myopia or high myopia were performed for all components at each school level, adjusted for age, parental myopia, parental education level, and SES. Components with significant associations in the analyses are marked with asterisks, daggers, and double daggers. * At the kindergarten level, a significant negative association was observed between component 5 and children’s myopia (adj. OR: 0.71, 95% CI: 0.51–0.98, *P* = 0.04). This component was majorly contributed by the parental attitude in regulating outdoor time and reading time. † At the elementary school level, a significant positive association was observed between component 1 and children’s myopia (adj. OR: 1.19, 95% CI: 1.05–1.34, *P* = 0.005), and a negative association was observed between component 3 and children’s myopia (adj. OR: 0.69, 95% CI: 0.61–0.77, *P* < 0.001). Component 3 was negatively affected by parental behavior in the medical control of myopia. ‡ At the high school level, no significant association was observed between the components and myopia in children. However, when we targeted moderate and high myopia (*n* = 963) subgroups, a negative association at a borderline significance level was observed between component 1 and children’s high myopia (adj. OR: 0.87, 95% CI: 0.75–1.0, *P* = 0.05)

PCA was conducted at each school level to further understand the detailed pattern of parental behavior. The weighting of each question contributing to each principal component is depicted on the heatmap in Fig. 2. The first component was majorly contributed by questions about parental behavior in daily care for their children, including maintaining reading postures and interrupting near work time, among others. The second component consisted of questions about parental attitude in limiting children’s time spent on electronic device use. The weighting patterns of the first two components were consistent across school levels. Some components were associated with children’s myopia or high myopia in multivariate logistic regression analyses, as shown in Fig. 2. In general, the associations were consistent with the results of the analysis of the total behavior score.

## Discussion

By analyzing 3845 completed questionnaires from the latest schoolchildren’s myopia survey in Taiwan, we found several associations between parental behavior and children’s myopia. First, the strength of parental behavior of children’s myopia prevention and control showed a decreasing trend that followed children’s age. Second, parental behavior and children’s myopic status may have a reciprocal effect. Third, parental behavior influences children’s daily activities, especially in the time spent on their near work activities and electronic device use at an early age.

Contrary to the positive association between beneficial parental behavior rate and degree of myopia in children found in our study, Zhou et al. found that parents’ attitudes and behaviors toward children’s visual care were associated with a lower risk of myopia in children [13]. They suggested that parental behavior influences children’s behavior regarding eye care, thus reducing the risk of myopia. The explanation for our finding may be the reciprocal effect between children’s myopia and parental behavior. It is possible that children’s myopic status may strengthen parents’ eye care behavior if they are aware of and recognize it as a health concern. The positive association between beneficial parental behavior and children’s myopia may reflect the effect of school-based vision surveillance system in Taiwan. In Taiwan, there are mandatory yearly vision examinations for every child from 4 years of age. In elementary school, children with uncorrected visual acuity of less than 20/20 are advised to consult an eye care professional. Subsequently, parents receive a notification about how to conduct beneficial behaviors toward myopia control and treatment options for myopia. We observed that parents of children with moderate myopia presented a higher rate of beneficial parental behavior than children with mild or without myopia (Fig. 1). Additionally, we observed that parents’ acceptance of medical treatment was significantly associated with children’s myopia status in the PCA at the elementary school level (Fig. 2). Therefore, we speculated that routine vision checks that effectively detect children’s vision problem may increase parents’ awareness and strengthen their behaviors for children’s eye care, although longitudinal studies are necessary to elucidate the causal relationships.

Another explanation is that parental behavior toward eye care has a limited protective effect on myopia development among children in Taiwan. An educational system involving intensive reading starting in early childhood in Taiwan is one of the key factors for the increased prevalence of myopia over generations for the whole population [14, 15]. Extremely high educational pressure and extended extracurricular learning at cram schools from a young age in East Asian societies result from a competitive school entrance system. In our study, we found that the prevalence of beneficial parental behavior reduced gradually from kindergarten to high school. We also found that parental behavior had minor impact on children’s outdoor activity time. One of the proposed explanations is that the concept of the protective role of outdoor activities on myopia prevention was introduced in just recent decade [7] and was unfamiliar to the parents of high schoolers. Another possible explanation is that when families are operating within the context of a school system that is highly competitive from an early age, the range of reasonable choices for parents is inevitably restricted, and parents’ ability to get their children more involved in outdoor activities is more constrained. Therefore, the preventive effect of beneficial parental behavior in children’s myopia development is incrementally counteracted after a few years of extensive educational pressures and cannot be observed in our cross-sectional study.

Our finding in the association between beneficial parental behavior and children’s myopia is likely to be a phenomenon unique to Taiwan, and probably other East and Southeast Asian countries with developed myopia epidemics and vigorous vision surveillance systems. It may not be observed in Western populations because they have a lower myopia incidence and a less academically competitive educational system. However, with the expected rising of global myopia prevalence, our finding may add new information in understanding the relationship between parental behavior and children’s myopia and help to improve the strategies in myopia control. In Taiwan, the effect of beneficial parental behavior may not be strong enough to concur the overwhelming environmental impact on myopia development because it is difficult for parents to make choices that effectively prevent their children from myopia development. Therefore, an education reform that substantially reduces academic loads in young children is of primary importance. Moreover, school-based programs promoting children’s outdoor activity should also be implemented due to the limited influence of parental behavior on children’s time outdoors [2, 7]. Finally, a well-functioning surveillance system for children’s vision that effectively inform the parents about children’s myopic status may help enhancing the beneficial parental behavior.

Although the association between beneficial parental behavior and children’s myopia prevention at the elementary school level was not observed in this study, we found that beneficial parental behavior was associated with less high myopia in high school children. High myopia represents the long-term outcome of unfavorable myopia progression [16]. Genetic predisposition, prolonged near work time, lack of active rest during the study, reduced outdoor activities, and inadequate sleeping time were all reported risk factors for myopia progression and high myopia [16–20]. Digital screen time, which contributes to further near workload, is also considered an important aggravating factor in myopia progression [6, 21]. Aside from genetic predisposition, these environmental factors are modifiable through the adjustment of children’s behaviors.

Many researchers have identified a strong relationship between parenting behaviors and children’s health status [22, 23]. The influence of parental behavior on children’s healthy behavior and medical compliance has been documented [8–10]. A systemic review found that parents’ encouragement increases children’s physical activity engagement and that less electronic device use of the parents is followed by minimized use in their children [11]. Our study also found a correlation between beneficial parental behavior and less total near work time or electronic device usage in children, especially at younger ages. We suggest that beneficial parental behavior toward children’s eye care may not reverse the incidence of myopia in Taiwan; however, myopia progression could be controlled by modifying children’s behaviors, thus preventing high myopia formation. Nevertheless, further longitudinal studies are necessary to elucidate the relationship between parental behaviors, children’s behavior patterns, and myopia status overall.

There are some limitations to our study. First, the cross-sectional design of our study could only demonstrate the association, rather than a causal relationship, between parental behavior and children’s myopia status. Second, the survey of parental behavior was based on questions quantified using scoring scales. The cut point of beneficial parental behavior was defined by the third quartile of the total score distribution, which requires further validation for appropriateness. PCA was performed to complement information loss through dichotomous grading of behavior scores. Third, the amplitude of myopia in both the father and mother is related to myopia in children in a dose-dependent manner [24]. In our study, information on parental myopia was obtained using a questionnaire without objective and quantitative measurements. Finally, our study focused only on regular parental behaviors toward children’s eye care. However, variable aspects of parenting, including parenting style, role modeling, self-efficacy, and perception of children’s health needs, may play a role in children’s health.

## Conclusion

In Taiwan, children with beneficial parental behavior do not have a lower risk of myopia. Multi-strategy approach incorporating parental behavior, education reform and school-based program are necessary to counteract children’s myopia. Awareness of children’s health condition may strengthen parents’ beneficial behaviors, which influence children’s activity pattern. Regular vision surveillance is recommended to promote better parental behavior toward children’s eye care, which is related to a reduced risk of high myopia development in the long run.

## Supplementary Information

**Additional file 1.** 

## Data Availability

1. Data availability: Most analytic results associated with this study are available in our previously published article.(https://doi.org/10.1016/j.ophtha.2020.07.017) 2. Data from this study are not available in a public archive. 3. De-identified data from this study will be made available upon emailing the corresponding author. 4. Analytic code availability: The analytic code from this study will be made available upon emailing the corresponding author. 5. Material availability: All materials used to conduct the study are available in the online supplemental material of our previously published article.

## Abbreviations

SES: Socioeconomic status
SE: Spherical equivalent
95% CI: 95% Confidence interval
OR: Odds ratio
PCA: Principal component analyses
