# Supplementary material for: Influence of parental behavior on myopigenic behaviors and risk of myopia: analysis of nationwide survey data in children aged 3 to 18 years
Source: BMC Public Health. 2022 Aug 30;22:1637. doi: 10.1186/s12889-022-14036-5 (PMC9426005; doi:10.1186/s12889-022-14036-5)
Supplement: Supplementary file 1 — Additional file 1. [file 12889_2022_14036_MOESM1_ESM.zip › mmc1.pdf]

Factors Related to Myopia Prevention and Treatment Questionnaire

Questionnaire Number:__________

Dear Parents:

High myopia is likely to cause complications leading to visual impairment or even blindness, which will seriously affect personal health and learning development. This survey aims to understand whether there are factors in your children's daily habits that tend to cause myopia. Therefore, please review your children's daily life related to their eye care in the past year, to find out the factors that may affect his vision.

The answers you reply will neither be judged as right or wrong nor affect the care your children will receive in the future. Please answer the question freely and honestly. If you have any questions, please contact us directly by phone (02-23123456#65184 or #62131), we will assist you immediately! Thank you for your support and cooperation!

We guarantee that the personal information of your children will be kept confidential.

Department of Ophthalmology, National Taiwan University Hospital

School Name:_________________ ____Grade____Class

Student Name:____________________ Gender: ☐①Male ☐②Female

Birthdate:_______________

Place of Residence:_______County, City______Township

☐I agree to answer the survey and fill it out clearly.

☐I do not agree to answer the survey and return the blank questionnaire.

Part I Background Information

I. Basic information of the schoolchild

1. The relationship between the person answering this questionnaire and the schoolchild:

   □①Parent-child relationship □②Grandparent-grandchild relationship □③ Other_______

2. The schoolchild was born: □① premature □② mature

   (1) The schoolchild was born by □① caesarean section □②vaginal delivery

   (2) The schoolchild was born at □① less than 30 weeks □②31-34 weeks □③35-36 weeks □④37-40 weeks □⑤41weeks and more.

   (3) The schoolchild weighed □① less than 1000 g □②1001-1500g □③1501-2500 g □④2501-4000 g □⑤over 4001 g when he/she was born.

3. Had the schoolchildren been breastfed?

   □① No □② Less than 3 months □③ 3~6 months □④ over 6 months.

4. Have the parents smoked when the mother was pregnant with the schoolchild?

   □①Yes, the mother has □②Yes, the father has □③Both have □④Neither has

5. If the mother has drunk alcohol when she was pregnant with the schoolchild, how often she drank?

   □① No □② From time to time at social occasions □③ Often

6. To understand the relationship between schoolchildren's vision and their living space, so please answer the following questions:

   (1) Currently, the schoolchild lives in □①a house □② an apartment, apartment building □③ Other_______

   (2) The area of the place the schoolchild lives in currently is about:

   □① less than 66 m^2 □② 67-132 m^2□③ 133-198 m^2□④ larger than 198 m^2

7. Is the schoolchild nearsighted?□① No (Please continue to answer question 11 □②Yes

8. When did the schoolchild become nearsighted? ________years old
9. Do the schoolchild wear corrective lenses and when he/she started to wear?

   ☐① Yes, since he/she was ________years old. ☐②No

10. Did the parents take the schoolchild to ophthalmology clinic for follow up check and treatment?

    ☐① No ☐② Yes Please write the number of visits to the clinic in the past year:___

11. The schoolchild usually goes to bed at ___pm and get up at__ am.
12. How many hours the schoolchild sleeps except at night?

    ☐①Less than 1 hour ☐②1~2 hours ☐③2~4 hours ☐④4 hours or more

13. When the schoolchild is sleeping, the lighting in the room is:

    ☐①The lights are all off ☐②A night light is on ☐③ Keep the lights on

14. The schoolchild's average academic achievement in the school is ranked

    ☐① top 20% ☐② 20%~40% ☐③ 40%~60% ☐④ 60%~80% ☐⑤ 80%~100%

15. When reading at home, the main source of light used by schoolchildren comes from:

    ☐①Desk lamp ☐②Ceiling lights ☐③Natural light through windows or outdoor light ☐④ Other___________

16. What is the main transportation way does the schoolchild uses to go to school and go home after school?

    ☐① Walking ☐② Riding a motorbike ☐③ Bus ☐④ MRT ☐⑤ Train ☐⑥ Car ☐⑦Other______

    (1) What does the schoolchild do on the way to school and home:

    ☐① Doesn't do anything else ☐② Reading ☐③ Writing ☐④ Chatting ☐⑤ Sleep ☐⑥ See scenes outside the window ☐⑦ Play smart phone, tablet ☐⑧Other_______

17. (1) The schoolchild eats breakfast____ times on average every week.
    (2) The schoolchild eats lunch____ times on average every week
    (3) The schoolchild eats dinner____ times on average every week

II. Parents' Background Information

To find out the relationship between your child's eyesight status and your vision, age, lifestyle, education and occupation, please answer the following questions:

1. How old the father was when the schoolchild was born.<br>☐①25 years old or younger<br>☐②26-35 years old<br>☐③36-45 years old<br>☐④ 46-55 years old<br>☐⑤56 years old or older	2. Father's education level<br>☐①Master’s degree or higher<br>☐②University or junior college graduate<br>☐③(Vocational) High school graduate or college non-completion<br>☐④Junior high school graduate<br>☐⑤Elementary school<br>☐⑥With no formal education	3. Father's eyesight status<br>☐①Not nearsighted or less than negative 0.5D<br>☐②Nearsighted<br>OD-Right:_____<br>OS-Left:_____<br>☐③ Farsighted<br>OD-Right:____<br>OS-Left:____<br>☐④Astigmatism<br>OD-Right:____<br>OS-Left:____<br>☐⑤ No idea
4. Father's degree of nicotine dependence<br>☐① Doesn’t smoke<br>☐②Smokes less than 5 cigarettes per day<br>☐③Smokes 5~10 cigarettes per day<br>☐④Smokes 11~20 cigarettes per day<br>☐⑤Smokes 21 cigarettes or more per day	5. Father's level of alcohol use<br>☐①Almost never drunk<br>☐②Just drink a little or drink at social occasions<br>☐③Moderate drinking (drink 2-4 days on average per week)<br>☐④Drink a lot (almost every day)	6. The schoolchild lives with his/her father<br>☐① almost every day<br>☐② 5-6 days a week<br>☐③ 3-4 days a week<br>☐④ 1-2 days a week<br>☐⑤ almost never

7. When the schoolchild was born his/her mother was<br>☐①25 years old or younger<br>☐②26-35 years old<br>☐③36-45 years old<br>☐④ 46-55 years old<br>☐⑤56 years old or older	8. Mother's education level<br>☐①Master's degree or higher<br>☐②University or junior college graduate<br>☐③(Vocational) High school graduate or college non-completion<br>☐④Junior high school graduate<br>☐⑤Elementary school<br>☐⑥With no formal education	9. Mother's eyesight status<br>☐①Not nearsighted or less than negative 0.5D<br>☐②Nearsighted<br>OD-Right:_____<br>OS-Left:_____<br>☐③ Farsighted<br>OD-Right:____<br>OS-Left:____<br>☐④Astigmatism<br>OD-Right:____<br>OS-Left:____<br>☐⑤ No idea
10. Mother's degree of nicotine dependence<br>☐① Doesn't smoke<br>☐②Smokes less than 5 cigarettes per day<br>☐③Smokes 5~10 cigarettes per day<br>☐④Smokes 11~20 cigarettes per day<br>☐⑤Smokes 21 cigarettes or more per day	11. Mother's level of alcohol use<br>☐①Almost never drunk<br>☐②Just drink a little or drink at social occasions<br>☐③Moderate drinking (drink 2-4 days on average per week)<br>☐④Drink a lot (almost every day)	12. 6. The schoolchild lives with his/her mother<br>☐① almost every day<br>☐② 5-6 days a week<br>☐③ 3-4 days a week<br>☐④ 1-2 days a week<br>☐⑤ almost never

13. Occupations of parents/guardians of the schoolchild: Please select the current occupation of the parents/guardians of the schoolchild according to the occupational category and items (if the parent or guardian has retired, write their occupation before retirement; if the parents are dead, please fill in the guardian's information.)			
Father	Mother	Guardian	Occupations
			Factory worker, hawker, farmer, fisherman, construction worker, janitor, handyman, temporary workers, maintenance worker, building caretaker, hired laborer, waiter
			Mechanic, plumber, shop assistant, small shop owner, retailers, salesperson, peasant, driver, tailor, chef, beautician, hairdresser, soldier (sergeant), typist, foreman, supervisor, working class
			Technician, assistant technical specialist, junior civil servant, officer, financial institution official, cashier, county and city councilor, township citizen representative, wholesaler, agent, contractor, junior officer, police, firefighter, secretary, scrivener, movie Or TV actor, petty bourgeoisie
			Primary or secondary school principal, primary or secondary school teacher, accountant, judge, lawyer, engineer, architect, associate civil servant, company chief, municipal councilman, manager, assistant managers, assistant president, deputy manager, middle range officer, police officer, writer, painter, musician, reporter or TV news reporter, head of an enterprise, managerial class
			College principal, (junior) college teacher, physician, justice, special or senior civil servant, legislator, member of Control Yuan, member of Examination Yuan, chairman, general manager, general, diplomat, bourgeoisie
			Homemaker, housekeeper

14. Average monthly gross income of the family in the past year:

□① Less than NT$ 30,000 □② NT$ 30,001-45,000 □③ NT$ 45,001-60,000 □④ NT$ 60,001-75,000 □⑤75,001-90,000 元 □⑥NT$90,001or more

15.Father's family origin: □①Indigenous people □②Hakka □③Hoklo Taiwanese □④ Mainlander □⑤New immigrant or foreigner, from ______

16.Mother's family origin: □①Indigenous people □②Hakka □③Hoklo Taiwanese □④ Mainlander □⑤New immigrant or foreigner, from______

Part II Factors related to myopia prevention and treatment for schoolchildren

I. Actions taken by parents to prevent and treat myopia for the schoolchild

In the past year, have you taken care of your child in the following ways to prevent him from developing myopia? Have you done this?

	Always	Usually	Occasionally	Seldom	Never
1. When I found that the lighting in the environment where the schoolchild was doing activities, I adjusted the lighting to be suitable. I have also installed lighting sources above, in front of or in the back left of the child's desk, so that he can have enough illumination when reading.	☐	☐	☐	☐	☐
2. (1) When the child went to kindergarten, I began to let him/her hold a pencil to practice writing.	☐	☐	☐	☐	☐
(2) When the child went to kindergarten, I began to let him/her practice calculation using an abacus.	☐	☐	☐	☐	☐
(3) When the child went to kindergarten, I began to let him/her practice mental arithmetic.	☐	☐	☐	☐	☐
(4) When the child went to kindergarten, I began to let him/her practice the piano.	☐	☐	☐	☐	☐
3. When I found that my child read or drew a picture in a poor sitting posture, sat at the desk with the desk surface higher than his/her chest, or his/her feet could not naturally touch the ground, I would help him make an adjustment immediately. I also remind him/her that the distance between his/her eyes and the book should be around 35 cm while he/she is reading.	☐	☐	☐	☐	☐
4. I would download the education apps to a smartphone or tablet for the child to play.	☐	☐	☐	☐	☐
5. I would download the game apps to a smartphone or tablet for the child	☐	☐	☐	☐	☐

to play.					
6. While the child is reading a book, drawing a picture, using a smartphone, tablet, or computer, or watching TV, I would interrupt him every 30-40 minutes, and let him/her look far and rest for 10 minutes.	☐	☐	☐	☐	☐
7. When I found that a book or a picture book poorly printed, with characters too small, or surface reflective, I would forbid him/her from reading it.	☐	☐	☐	☐	☐
8. I asked the child to go to bed at around 10 o'clock pm.	☐	☐	☐	☐	☐
9. I let the child have a balanced diet every day.	☐	☐	☐	☐	☐
10. When I found that the child squinted at something, I would want to know why he/she squinted. If the child felt his/her eyesight was blurred or complained that he/she felt uncomfortable, I would take him to a doctor immediately.	☐	☐	☐	☐	☐
11. I would help the child administer mydriatic before he/she go to bed to slow the progression of myopia. (1) The drug name of the mydriatic: _______, with a concentration of ______	☐	☐	☐	☐	☐
12. I would let the schoolchild wear Orthokeratology lenses to slow the progression of myopia.	☐	☐	☐	☐	☐

II. Parents' assessments of child's visual behaviors

1. I would accompany my schoolchild to exercise outdoors at least___hours during the day.
2. I limit the time for the schoolchild to watch TV, no more than ___ hours per day.
3. I let the schoolchild spend ____ hours in reading, drawing, and doing homework at home.
4. Do you agree that parents should allow their schoolchildren to use a smartphone or tablet?

   ☐① Yes (Please continue to answer the second question) ☐② No

   (1) What product a schoolchild can use?

   ☐①Smartphone ☐② Tablet ☐③Desktop Computer ☐④ All of them

   (2) I restrict the child from using a desktop computer for ________ hours on weekdays and ________ hours on holidays.

   (3) I restrict the child from using a smartphone for ________ hours on weekdays and ________ hours on holidays.

   (4) I restrict the child from using a tablet for ________ hours on weekdays and ________

hours on holidays.

5. Do you agree that parents should allow their schoolchildren to watch TV?☐①Yes (Please continue to answer the second question) ☐②No

   (2) If you agree, you allow the child to watch ____hours of TV on weekdays and _____hours on holidays.

6. I let the child go to the cramming school and attend the courses in ☐①Chinese (including composition) ☐②English ☐③ Mathematics (multiple answer question)

   (2) A total of ______ hours of tutoring on weekdays, a total of ________ hours on weekend

III. Schoolchild's personal visual behaviors

1. Please write down what activities you did in your daily life in the past year, so that we can estimate the burden of your vision during the year. The more clearly you answer the questions, the more helpful for us to understand your vision problems.

A. In the past year, how was the child's time divided among the activities after school on weekdays?

Activities	Length of Time
(1) Write homework	☐①None ☐②about_____min ☐③ No idea
(2) Tutoring-	
Chinese (including composition)	☐①None ☐②about_____min ☐③ No idea
English	☐①None ☐②about_____min ☐③No idea
Mathematics	☐①None ☐②about_____min ☐③ No idea
(3) Extracurricular reading, art and craft	☐①None ☐②about_____min ☐③ No idea
(4) Use the 3C products ☐①Desktop Computer ☐②Tablet ☐③Smartphone	
1. What was the type of smartphone apps that has been most frequently used? (Please sequence them.)	
☐①Games ☐②Social communications ☐③Audio and video media ☐④Online shopping	
2. What was the type of tablet apps that has been most frequently used? (Please sequence them.)	
☐①Games ☐②Social communications ☐③Audio and video media ☐④Online shopping	
(5) Use console to play video games	☐①None ☐②about___min ☐③ No idea
(6) Watch TV	☐①None ☐②about___min ☐③ No idea
(7) Use a desktop computer	☐①None ☐②about___min ☐③ No idea
(8) Play a smartphone	☐①None ☐②about___min ☐③No idea
(9) Play a tablet	☐①None ☐②about___min ☐③No idea
(10) Outdoor sports	☐①None ☐②about___min ☐③ No idea
(11) Indoor sports (such as table tennis, dancing)	☐①None ☐②about___min ☐③No idea

(12) Indoor activities - (such as cooking, bridge, chess, board game)	☐①None ☐②about___min ☐③ No idea
(13) Outdoor activities - (e.g. picnic, outing)	☐①None ☐②about___min ☐③No idea

(14)
1. The total number of hours the schoolchild stayed outdoors on average every school day after school was about ______ minutes.
2. What hours did you stay outdoors more often? (multiple answer question)

☐①Before 8 am ☐②12 noon~2 pm ☐③ 2 pm~4 pm ☐④ 4pm~7 pm ☐⑤ After 7 pm

(15) Other	☐①None ☐②about___min ☐③ No idea

B. In the past year, how has the child's time been divided among the activities on weekends?

Activities	Length of Time
(1) Write homework	☐①None ☐②about___min ☐③ No idea
(2) Tutoring- ①Chinese (including composition)	☐①None ☐②about___min ☐③ No idea
- ②English	☐①None ☐②about___min ☐③No idea
- ③Mathematics	☐①None ☐②about___min ☐③No idea
(3) Extracurricular reading, art and craft	☐①None ☐②about___min ☐③ No idea

(4) Use the 3C products ☐①Desktop Computer ☐②Tablet ☐③Smartphone

1. What was the type of smartphone apps that has been most frequently used? (Please sequence them.)

   ☐①Games ☐②Social communications ☐③Audio and video media ☐④Online shopping

2. What was the type of tablet apps that has been most frequently used? (Please sequence them.)

   ☐①Games ☐②Social communications ☐③Audio and video media ☐④Online shopping

(5) What hours did you use the tablet more often? (multiple answer question)

☐① 6 am~8 am ☐② 8 am~12 noon ☐③ 12 noon~2 pm ☐④ 2 pm~6 pm ☐⑤ 6 pm-9 pm ☐⑥9 pm-12 pm

(6) What hours did you watch TV more often? (multiple answer question)

☐① 6 am~8 am ☐② 8 am~12 noon ☐③ 12 noon~2 pm ☐④ 2 pm~6 pm ☐⑤ 6 pm-9 pm ☐⑥9 pm-12 pm

(7) Use console to play video games	☐①None ☐②about___min ☐③ No idea
(8) Watch TV	☐①None ☐②about___min ☐③No idea
(9) Use a desktop computer	☐①None ☐②about___min ☐③ No idea
(10) Play a smartphone	☐①None ☐②about___min ☐③No idea
(11) Play a tablet	☐①None ☐②about___min ☐③ No idea

(12) Outdoor sports	☐①None ☐②about___min ☐③ No idea
(13) Indoor sports (such as table tennis, dancing)	☐①None ☐②about___min ☐③ No idea
(14) Indoor activities - (such as cooking, bridge, chess, board game)	☐①None ☐②about___min ☐③ No idea
(15) Outdoor activities - (e.g. picnic, outing)	☐①None ☐②about___min ☐③ No idea

(16)

1. The average number of hours the schoolchild spent outdoors on weekends is about ______ minutes.
2. What hours did you stay outdoors more often? (multiple answer question)

☐①Before 8 am ☐② 8 am~12 noon ☐③ 12 noon~2 pm ☐④ 2 pm~4 pm ☐⑤4 pm~7 pm ☐⑥After 7 pm

(17) Other	☐①None ☐②about___min ☐③ No idea

C. In the past year, how has the child's time been divided among the activities at recess at school on weekdays?

Activities	Length of Time
(1) Reading, reviewing, and writing homework	☐①None ☐②about___min ☐③ No idea
(2) Drawing, crafts	☐①None ☐②about___min ☐③ No idea
(3) Do exercise	☐①None ☐②about___min ☐③No idea
(4) Cleaning	☐①None ☐②about___min ☐③ No idea
(5) Playing cards, board games, chess	☐①None ☐②about___min ☐③ No idea
(6) Going outdoors to play and exercise	☐①None ☐②about___min ☐③ No idea
(7) Sleep, rest	☐①None ☐②about___min ☐③No idea
(8) Have a meal	☐①None ☐②about___min ☐③No idea
(9) Other:	☐①None ☐②about___min ☐③No idea

D. In the past year, how has the child's time been divided among the activities every day in summer and winter vacation?

Activities	Length of Time
(1) Write homework	☐①None ☐②about___min ☐③ No idea
(2) Tutoring- ①Chinese (including composition)	☐①None ☐②about___min ☐③ No idea
- ②English	☐①None ☐②about___min ☐③ No idea
- ③Mathematics	☐①None ☐②about___min ☐③ No idea

(3) Extracurricular reading, art and craft ☐①None ☐②about___min ☐③ No idea

(4) Use the 3C products ☐①Desktop Computer ☐②Tablet ☐③Smartphone

1. What was the type of smartphone apps that has been most frequently used? (Please sequence them.)

   ☐①Games ☐②Social communications ☐③Audio and video media ☐④Online shopping

2. What was the type of tablet apps that has been most frequently used? (Please sequence them.)

   ☐①Games ☐②Social communications ☐③Audio and video media ☐④Online shopping

(5) What hours did you use the tablet more often? (multiple answer question)

☐① 6 am~8 am ☐② 8 am~12 noon ☐③ 12 noon~2 pm ☐④ 2 pm~6 pm ☐⑤ 6 pm-9 pm ☐⑥9 pm-12 pm

(6) What hours did you watch TV more often? (multiple answer question)

☐① 6 am~8 am ☐② 8 am~12 noon ☐③ 12 noon~2 pm ☐④ 2 pm~6 pm ☐⑤ 6 pm-9 pm ☐⑥9 pm-12 pm

(7) Use console to play video games ☐①None ☐②about___min ☐③ No idea

(8) Watch TV ☐①None ☐②about___min ☐③No idea

(9) Use a desktop computer ☐①None ☐②about___min ☐③ No idea

(10) Play a smartphone ☐①None ☐②about___min ☐③ No idea

(11) Play a tablet ☐①None ☐②about___min ☐③ No idea

(12) Outdoor sports ☐①None ☐②about___min ☐③ No idea

(13) Indoor sports (such as table tennis, dancing) ☐①None ☐②about___min ☐③ No idea

(14) Indoor activities - (such as cooking, bridge, chess, board game) ☐①None ☐②about___min ☐③No idea

(15) Outdoor activities - (e.g. picnic, outing) ☐①None ☐②about___min ☐③No idea

(16)

1. The total number of hours the schoolchild stayed outdoors during the winter vacation was about ______ minutes.
2. What hours did you stay outdoors more often? (multiple answer question)

   ☐①Before 8 am ☐② 8 am~12 noon ☐③ 12 noon~2 pm ☐④ 2 pm~4 pm ☐⑤4 pm~7 pm ☐⑥After 7 pm

3. The total number of hours the schoolchild stayed outdoors during the summer vacation was about ______ minutes.
4. What hours did you stay outdoors more often? (multiple answer question)

   ☐①Before 8 am ☐② 8 am~12 noon ☐③ 12 noon~2 pm ☐④ 2 pm~4 pm ☐⑤4 pm~7 pm ☐⑥After 7 pm

(17) Other ☐①None ☐②about___min ☐③ No idea

E. In the past year, the total hours the schoolchild spent in the outdoor activities of school physical education classes each week.

☐①None ☐②about___min ☐③No idea

All the questions are over, please check again whether you have answered all the questions. Thank you for your cooperation!
