# Supplementary material for: Influence of parental behavior on myopigenic behaviors and risk of myopia: analysis of nationwide survey data in children aged 3 to 18 years
Source: BMC Public Health. 2022 Aug 30;22:1637. doi: 10.1186/s12889-022-14036-5 (PMC9426005; doi:10.1186/s12889-022-14036-5)
Supplement: Supplementary file 1 — Additional file 1. [file 12889_2022_14036_MOESM1_ESM.zip › mmc2.pdf]

eMethods

The 1983 survey stratified 5 sociogeographical categories (metropolitan precincts, provincial cities, townships, villages, and aboriginal areas) in areas of Taiwan based on population, degree of urbanization, and level of education.[1] In total, 15 kindergartens, 16 primary schools, 16 junior high schools, 19 senior high schools, and 17 vocational schools (including the first-, second-, and third-year graduates belonging to a 5-year junior college program [age, 16–18 y]) were randomly selected from the strata defined by age, grade, and sex. A total of 9075 schoolchildren and 825 preschool children were randomly selected; among them, 4455 (49.1%) schoolchildren and 564 (68.4%) preschool children completed the examinations conducted in the survey and included in the study.

In 1986, the same sampling method was applied, and 18 primary schools, 19 junior high schools, 31 senior high schools, and 33 vocational schools were randomly selected. A total of 10 500 (10 500/11 000, 95.5%) schoolchildren participated in the study.[2] Through the same sampling method in 1990, 14 primary schools, 7 junior high schools, 9 senior high schools, and 8 vocational schools were randomly selected, and a total of 8667 (8667/9500, 91.2%) schoolchildren participated in the study.[3]

In 1995, in an attempt to stratify the samples more effectively, we adopted the probability proportional to size sampling method with replacement based upon the developmental gradings of areas in Taiwan. Urbanization was grouped into 10 distinct strata including 2 metropolitan precincts (Taipei City and Kaohsiung City), provincial cities, developing areas, industrial areas, service business areas, combination areas, remote areas, hilly areas, and aboriginal areas.[4] There were 37 primary schools, 22 junior high schools, 6 senior high schools, and 11 vocational schools selected, and a total of 11 178 (11 178/11 882, 94%) school students participated in this survey, including 5676 boys and 5502 girls.[4]

In 2000, the same stratified sampling method was employed.[5] Forty-one primary schools, 39 junior high schools, 13 senior high schools, and 13 vocational schools were selected and 10 889 (10 889/11 875, 91.7%) schoolchildren participated in the study, including 5664 boys and 5225 girls.[5,6]

In 2005, 7 district urbanization levels were considered (metropolitan precincts, provincial cities, emerging towns, general towns, aging towns, agricultural towns, and remote towns) based on a new categorization system modified to comply with the development in Taiwan's regions.[7] There were 28 primary schools, 30 junior high schools, 8 senior high schools, and 10 vocational schools selected, and 11 656 (11 656/18 036, 64.6%) schoolchildren participated in the study, comprising 5390 boys and 6260 girls.[8]

In 2010, we used the same stratification system as in 2005. Because of the limited budget in this survey, only 27 elementary schools and 6075 (6075/6857, 88%) students, including 3076 boys and 2999 girls, were examined.[9]

In the latest survey conducted in 2016-2017, 3 district urbanization levels (metropolitan precincts, provincial cities, and other areas) were applied to comply with the development in Taiwan. Twenty-three primary schools, 20 junior high schools, 23 senior high schools, and 35 kindergartens were selected. A total of 10 000 students aged 3 to 18 years were selected, and 7348 (73.48%), including 3931 boys and 3417 girls, participated in the study (Table 2).

References

1. Lin LL, Shih YF, Chen CJ, Hung PT, Hou PK. Epidemiologic study of ocular refraction among schoolchildren in Taiwan in 1983. Taipei, Taiwan: Executive Yuan, Taiwan. 1983.
2. Lin LL, Chen CJ, Hung PT, Ko LS. Nation-wide survey of myopia among schoolchildren in Taiwan, 1986. *Acta ophthalmologica Supplement.* 1988;185:29-33.
3. Lin LL, Shih YF, Chen CJ, Hung PT, Hou PK. Epidemiologic study of ocular refraction among schoolchildren in Taiwan in 1990. *Annual Reports of Department of Health, Executive Yuan, Taiwan.* 1990.
4. Lin LL, Shih YF, Tsai CB, et al. Epidemiologic study of ocular refraction among schoolchildren in Taiwan in 1995. *Optometry and vision science : official publication of the American Academy of Optometry.* 1999;76(5):275-281.
5. Lin LL, Shih YF, Hsiao CK, Chen CJ, Lee LA, Hung PT. Epidemiologic study of the prevalence and severity of myopia among schoolchildren in Taiwan in 2000. *Journal of the Formosan Medical Association = Taiwan yi zhi.* 2001;100(10):684-691.
6. Lin LL, Shih YF, Hsiao CK, Chen CJ. Prevalence of myopia in Taiwanese schoolchildren: 1983 to 2000. *Annals of the Academy of Medicine, Singapore.* 2004;33(1):27-33.
7. Liu CY, Hung YT, Chuang YL, et al. Incorporating development stratification of Taiwan townships into sampling design of large scale health interview survey (in Chinese). *Journal of Health Management.* 2006;4:1:1-22.
8. Lin LL, Shih YF, Hsiao CH. Epidemiologic study of ocular refraction among 6-18 year old schoolchildren in Taiwan in 2005. *Executive Yuan, Health Promotion Administration, Taiwan.* 2005.
9. Shih YF, Lin LL. Epidemiologic study of ocular refraction among preschoolchildren in Taiwan in 2010. *Executive Yuan, Health Promotion Administration, Taiwan.* 2010.
