# Supplementary material for: Influence of parental behavior on myopigenic behaviors and risk of myopia: analysis of nationwide survey data in children aged 3 to 18 years
Source: BMC Public Health. 2022 Aug 30;22:1637. doi: 10.1186/s12889-022-14036-5 (PMC9426005; doi:10.1186/s12889-022-14036-5)
Supplement: Supplementary file 1 — Additional file 1. [file 12889_2022_14036_MOESM1_ESM.zip › mmc3.pdf]

eTable 1. Stratified sampling methods and sampling results of 8 consecutive surveys from 1983 to 2017.

Years	Sampling method	Stratification	Response rate	Levels of school (number)	Sample size (boys, girls)	Population size (boys, girls)
1983	Stratified systematic cluster sampling	5 urbanizations[a]	50.7%	High school (36)	1,816 (915, 901)	581,273 (299,415, 281,858)
				Junior high school (16)	833 (411, 422)	1,082,358 (562,249, 520,109)
				Elementary school (16)	1,806 (906, 900)	2,226,699 (1,143,746, 1,082,953)
				Kindergarten (15)	564 (297, 267)	193,744 (102,377, 91,367)
1986	Stratified systematic cluster sampling	5 urbanizations[a]	95.5%	High school (64)	3,593 (1962, 1631)	616,541 (314,177, 302,364)
				Junior high school (19)	2,376 (1296, 1080)	1,062,226 (547,774, 514,452)
				Elementary school (18)	4,531 (2402, 2129)	2,321,700 (1,195,470, 1,126,230)
1990	Stratified systematic cluster sampling	5 urbanizations[a]	91.2%	High school (17)	2,252 (1203, 1049)	642,597 (311,747, 330,850)
				Junior high school (7)	1,973 (884, 1089)	1,125,238 (576,307, 548,931)
				Elementary school (14)	4,442 (2210, 2232)	2,384,801 (1,228,861, 1,155,940)
1995	Probability proportional to size sampling with replacement	10 urbanizations[b]	94.0%	High school (17)	2,210 (1069, 1141)	769,670(375,151, 394,519)
				Junior high school (22)	3,017 (1520, 1497)	1,177,352 (604,314, 573,038)
				Elementary school (37)	5,951 (3087, 2864)	2,032,361 (1,050,538, 981,823)
2000	Probability proportional to size sampling with replacement	10 urbanizations[b]	91.7%	High school (26)	2,475 (1197,1278)	798,825 (400,954, 397,871)
				Junior high school (39)	2,867 (1552,1315)	957,209 (493,262, 463,947)
				Elementary school (41)	5,547 (2915,2632)	1,927,179 (1,005,536, 921,643)
2005		7 urbanizations[c]	64.6%	High school (18)	1,874 (789, 1,085)	735,794 (383,347, 352,447)
				Junior high school (30)	3,914 (1,914, 2,000)	956,927 (500,083, 456,844)

	Probability proportional to size sampling with replacement			Elementary school (28)	5,868 (2,687, 3,181)	1,883,533 (980,531, 903,002)
2010	Probability proportional to size sampling with replacement	7 urbanizations[c]	88%	Elementary school (27)	6,075 (3,076, 2,999)	1,593,398 (831,989, 761,409)
2016	Probability proportional to size sampling with replacement	3 urbanizations[d]	73.48%	High school (23)	1,372 (826, 546)	719,920 (381,864, 338,056)
				Junior high school (20)	1,467 (750, 717)	800,775 (418,132, 382,643)
				Elementary school (23)	2,684 (1,386, 1,298)	1,248,538 (653,570, 594,968)
				Kindergarten (35)	1,825 (969, 856)	444,457 (233,464, 210,993)

High school = senior high school or vocational school; [a] = metropolitan precincts, provincial cities, townships, villages, and aboriginal areas; [b] = 2 metropolitan precincts (Taipei City and Kaohsiung City), provincial cities, developing areas, industrial areas, service business areas, combination areas, remote areas, hilly areas, and aboriginal areas; [c] = metropolitan precincts, provincial cities, emerging towns, general towns, aging towns, agricultural towns, and remote towns; [d] = metropolitan precincts, provincial cities, and other area
