# Supplementary material for: Influence of parental behavior on myopigenic behaviors and risk of myopia: analysis of nationwide survey data in children aged 3 to 18 years
Source: BMC Public Health. 2022 Aug 30;22:1637. doi: 10.1186/s12889-022-14036-5 (PMC9426005; doi:10.1186/s12889-022-14036-5)
Supplement: Supplementary file 1 — Additional file 1. [file 12889_2022_14036_MOESM1_ESM.zip › mmc4.pdf]

eTable 2. Distribution of urbanization level and school grade in the 2016-2017 survey and the population in Taiwan.

			Sample size	Percentage (%)	Population	Percentage (%)
Kindergarten children	Urbanization level	Metropolitan precincts	957	52.44	206,652	46.70
		Provincial cities	716	39.23	192,873	43.59
		Other areas	152	8.33	42,959	9.71
	School grade	Junior class	300	16.44	111,808	25.27
		Middle Class	698	38.24	144,526	32.66
		Senior class	827	45.32	186,150	42.07
		Total	1825	100	442,484	100
Elementary schoolchildren	Urbanization level	Metropolitan precincts	1346	50.15	633,052	50.70
		Provincial cities	910	33.90	497,729	39.87
		Other areas	428	15.95	117,757	9.43
	School grade	Grade 1	448	16.69	197,528	15.82
		Grade 2	483	18.00	198,185	15.87
		Grade 3	440	16.39	201,218	16.12
		Grade 4	400	14.90	208,255	16.68
		Grade 5	459	17.10	214,053	17.14
		Grade 6	454	16.92	229,299	18.37
		Total	2684	100	1,248,538	100
Junior high schoolchildren	Urbanization level	Metropolitan precincts	570	38.83	424,074	52.96
		Provincial cities	711	48.43	306,750	38.31
		Other areas	187	12.74	69,951	8.73
	School grade	Grade 7	542	36.92	242,105	30.23
		Grade 8	515	35.08	274,185	34.24
		Grade 9	411	28.00	284,485	35.53
		Total	1468	100	800,775	100
Senior high and vocational schoolchildren	Urbanization level	Metropolitan precincts	704	51.31	428,812	59.56
		Provincial cities	526	38.34	252,313	35.05
		Other areas	142	10.35	38,795	5.39
	School grade	Grade 10	453	33.02	233,938	32.49
		Grade 11	559	40.74	236,417	32.84
		Grade 12	360	26.24	249,565	34.67
		Total	1372	100	719,920	100
