# Supplementary material for: Influence of parental behavior on myopigenic behaviors and risk of myopia: analysis of nationwide survey data in children aged 3 to 18 years
Source: BMC Public Health. 2022 Aug 30;22:1637. doi: 10.1186/s12889-022-14036-5 (PMC9426005; doi:10.1186/s12889-022-14036-5)
Supplement: Supplementary file 1 — Additional file 1. [file 12889_2022_14036_MOESM1_ESM.zip › mmc5.pdf]

eTable 3. Devices used to measure cycloplegic refraction and eyeball axial length in each survey.

Survey year	Autorefractometer	A-scan ultrasonography
1983	Canon® R-10	Sonomed® A-1000
1986	Canon® R-10	Sonomed® A-1000
1990	Canon® R-10	Sonomed® A-1000
1995	Topcon® RK-3000	Sonomed® A-1500
2000	Topcon® RK-3000	Sonomed® A-1500
2005	Topcon® KR-1	Sonomed® 5500A
2010	Topcon® KR8- 900	Sonomed® 5500A
2016	Topcon® KR-800S	Accutome®
