# Supplementary material for: Influence of parental behavior on myopigenic behaviors and risk of myopia: analysis of nationwide survey data in children aged 3 to 18 years
Source: BMC Public Health. 2022 Aug 30;22:1637. doi: 10.1186/s12889-022-14036-5 (PMC9426005; doi:10.1186/s12889-022-14036-5)
Supplement: Supplementary file 1 — Additional file 1. [file 12889_2022_14036_MOESM1_ESM.zip › mmc6.pdf]

eTable 4. Demographic data and distribution of schoolchildren's daily activities in the 2005 and 2016 survey based on valid questionnaire responses. (Definition of myopia: spherical equivalent of ≤−0.25 D)

Year of survey	2005 (n=4005) Myopia (≤-0.25D) (n=3275)	No myopia (n=730)	p value	2016 (n=3190) Myopia (≤-0.25D) (n=1692)	No myopia (n=1498)	p value
	mean ±SD, n (%)			mean±SD, n (%)		
Age (year)*	15.3±1.7	14.8±1.6	<0.0001	12.6±3.7	6.4±3.3	<0.0001
Sex (female)	1875(57.3)	382(52.3)	0.02	834 (49.3)	739 (49.3)	0.98
Daily sleeping time (hour)						
< 9 hours	2964(90.5)	624(85.5)	<0.0001	1248(73.8)	376(25.1)	<0.0001
≥ 9 hours	311(9.5)	106(14.5)		444(26.2)	1122(74.9)	
Time spent on near work (minute per day)						
< 60	1093(33.4)	293(40.1)	<0.0001	299(17.7)	630(42.0)	<0.0001
60 – 180	1869(57.1)	392(53.7)		445(26.3)	609(40.7)	
≥ 180	313(9.5)	45(6.2)		948(56.0)	259(17.3)	
Daily outdoor exercise (yes)	1503(45.9)	401(54.9)	<0.0001	N/A	N/A	
Time spent on outdoor activity (minute per day)						
< 60	N/A	N/A		386(22.8)	332(22.2)	0.66
≥ 60				1306(77.2)	1166(77.8)	

*In 2005, the questionnaires were collected primarily from junior and senior high school children. In 2016, the questionnaires were collected from all school grades, including kindergarten
