# Supplementary material for: Influence of parental behavior on myopigenic behaviors and risk of myopia: analysis of nationwide survey data in children aged 3 to 18 years
Source: BMC Public Health. 2022 Aug 30;22:1637. doi: 10.1186/s12889-022-14036-5 (PMC9426005; doi:10.1186/s12889-022-14036-5)
Supplement: Supplementary file 1 — Additional file 1. [file 12889_2022_14036_MOESM1_ESM.zip › mmc7.pdf]

eTable 5. Demographic data and distribution of schoolchildren's daily activities in the 2005 and 2016 survey based on valid questionnaire responses. (Definition of myopia: spherical equivalent of ≤−0.50 D)

Year of survey	2005 (n=4005) Myopia (≤-0.50D) (n=3275)	No myopia (n=730)	p value	2016 (n=3190) Myopia (≤-0.50D) (n=1624)	No myopia (n=1566)	p value
	mean±SD, n (%)			mean±SD, n (%)		
Age (year)*	15.3±1.7	14.9±1.6	<0.0001	12.9±3.6	6.7±3.5	<0.0001
Sex (female)	1732(57.7)	525(52.2)	0.02	779 (48.8)	794 (49.8)	0.558
Daily sleeping time (hour)						
< 9 hours	2729(91.0)	859(85.5)	<0.0001	1195(76.5)	429(26.4)	<0.0001
≥ 9 hours	271(9.0)	146(14.5)		368(23.5)	1198(73.6)	
Time spent on near work (minute per day)						
< 60	973(32.4)	413(41.1)	<0.0001	264(16.9)	665(40.9)	<0.0001
60 – 180	1730(57.7)	531(52.8)		400(25.6)	654(40.2)	
≥ 180	297(9.9)	61(6.1)		899(57.5)	308(18.9)	
Daily outdoor exercise (yes)	1365(45.5)	539(53.6)	<0.0001	N/A	N/A	
Time spent on outdoor activity (minute per day)						
< 60	N/A	N/A		367(21.6)	351(23.5)	0.197
≥ 60				1196(78.4)	1276(76.5)	

*In 2005, the questionnaires were collected primarily from junior and senior high school children. In 2016, the questionnaires were collected from all school grades, including kindergarten.
