# Supplementary material for: Influence of parental behavior on myopigenic behaviors and risk of myopia: analysis of nationwide survey data in children aged 3 to 18 years
Source: BMC Public Health. 2022 Aug 30;22:1637. doi: 10.1186/s12889-022-14036-5 (PMC9426005; doi:10.1186/s12889-022-14036-5)
Supplement: Supplementary file 1 — Additional file 1. [file 12889_2022_14036_MOESM1_ESM.zip › mmc8.pdf]

eTable 6. The univariate and multivariate risk factor analysis of schoolchildren's behavior for myopia (≤ -0.50D) from 2005 and 2016 survey samples with valid questionnaire response (2005: n=4005; 2016: n=3190)

	Univariate Analysis, Crude Odds Ratio (95% Confidence Interval)		Multivariate Analysis, Adjusted Odds Ratio (95% Confidence Interval)	
Year of survey	2005	2016	2005	2016
Age (per 1 year increase)	1.17(1.12,1.22)*	1.48 (1.42,1.52)*	1.18 (1.13,1.24)*	1.42 (1.38,1.47)*
Female sex	1.25 (1.08,1.44)†	1.04 (0.91,1.20)	1.06 (0.91,1.23)	1.09 (0.90,1.32)
Sleeping time (hour per day)				
< 9	reference	Reference	reference	reference
≥ 9	0.58 (0.47,0.73)*	0.11 (0.09,0.13)*	0.68 (0.55,0.85) ‡	0.86 (0.68,1.09)
Time spent on near work activities (minute per day)				
≥ 180	reference	Reference	reference	reference
60 – 180	0.67 (0.50,0.90)‡	0.21 (0.17,0.25)*	0.68 (0.51,0.91)†	0.66 (0.53,0.83)*
< 60	0.48 (0.36,0.65)*	0.15 (0.12,0.18)*	0.44 (0.32,0.59)*	0.51 (0.39,0.66)*
Daily outdoor exercise§				
No	reference		Reference	
Yes	0.72 (0.63,0.83)*		0.79 (0.68,0.92)‡	
Time spent on outdoor activities (minute per day)§				
≥ 60		reference		reference
< 60		1.12 (0.95,1.32)		0.97 (0.77,1.24)

D, diopter; OR, odds ratio; CI, confidence interval

* *P* < 0.001.

† *P* < 0.05.

‡ *P* < 0.01.

§For outdoor activity, dichotomous question was used in 2005 questionnaire, while the estimated time was asked in 2016 questionnaire.
