# Supplementary material for: Influence of parental behavior on myopigenic behaviors and risk of myopia: analysis of nationwide survey data in children aged 3 to 18 years
Source: BMC Public Health. 2022 Aug 30;22:1637. doi: 10.1186/s12889-022-14036-5 (PMC9426005; doi:10.1186/s12889-022-14036-5)
Supplement: Supplementary file 1 — Additional file 1. [file 12889_2022_14036_MOESM1_ESM.zip › mmc9.pdf]

eTable 7. Weighted myopia prevalence in schoolchildren from 2016 survey by different definition of myopia and high myopia.

definition			Myopia prevalence (%)			High myopia prevalence (%)		
			≤ -0.25D	≤ -0.50D	p value	≤ -5.0D	≤ -6.0D	p value
School level	Grade	Mean age						
Kindergarten	Junior	3.21 ± 0.53	11.06	6.94	<0.0001	0.43	0.43	NA
	Middle	4.18 ± 0.48	9.73	7.38	0.0005	0.00	0.00	NA
	Senior	5.14 ± 0.39	12.11	8.98	<0.0001	0.52	0.52	NA
Elementary school	1	6.40 ± 0.50	25.41	19.76	<0.0001	1.18	0.71	0.48
	2	7.44 ± 0.69	45.31	38.73	<0.0001	1.41	0.94	0.48
	3	8.44 ± 0.57	50.00	43.29	<0.0001	1.62	0.23	0.04
	4	9.40 ± 0.50	57.14	52.68	<0.0001	4.24	1.56	0.001
	5	10.41 ± 0.51	70.00	62.17	<0.0001	6.74	2.61	<0.0001
	6	11.43 ± 0.51	76.67	70.59	<0.0001	10.34	4.26	<0.0001
Junior high school	7	12.56 ± 0.50	85.36	81.76	0.0002	15.32	9.23	<0.0001
	8	13.49 ± 0.51	89.07	85.29	<0.0001	19.48	11.53	<0.0001
	9	14.54 ± 0.59	92.90	89.25	<0.0001	28.02	15.36	<0.0001
Senior high school	10	15.52 ± 0.51	88.12	86.32	0.013	27.13	16.82	<0.0001
	11	16.53 ± 0.57	91.78	89.11	0.001	31.56	20.00	<0.0001
	12	17.47 ± 0.53	90.34	87.18	0.0003	35.71	24.16	<0.0001

All prevalence data is the adjusted value calculated by giving weighting in each stratum of urbanization level based on whole population data.

NA, not available; D, diopter.

McNemar’s test was used to compare the prevalence rate by different definition of myopia and high myopia.
